# Deep Learning for Gas Sensing via Infrared Spectroscopy

**DOI:** 10.3390/s24061873

**Published:** 2024-03-14

**Authors:** M. Arshad Zahangir Chowdhury, Matthew A. Oehlschlaeger

**Affiliations:** Department of Mechanical, Aerospace, and Nuclear Engineering, Rensselaer Polytechnic Institute, 110 Eighth St., Troy, NY 12180, USA

**Keywords:** infrared absorption spectroscopy, deep learning, classification, speciation, gas sensing, trace gas detection, atmospheric detection

## Abstract

Deep learning methods, a powerful form of artificial intelligence, have been applied in a number of spectroscopy and gas sensing applications. However, the speciation of multi-component gas mixtures from infrared (IR) absorption spectra using deep learning remains to be explored. Here, we propose a one-dimensional deep convolutional neural network gas classification model for the identification of small molecules of interest based on IR absorption spectra in flexible user-defined frequency ranges. The molecules considered include ten that are of interest in the atmosphere or in industrial and environmental processes: water vapor, carbon dioxide, ozone, nitrous oxide, carbon monoxide, methane, nitric oxide, sulfur dioxide, nitrogen dioxide, and ammonia. A simulated dataset of IR absorption spectra for mixtures of these molecules diluted in air was generated and used to train a deep learning model. The model was tested against simulated spectra containing noise and was found to provide speciation predictions with accuracy from 82 to 97%. The internal operation of the model was investigated using class activation maps that illustrate how the model prioritizes spectral information for classification. Finally, the model was demonstrated for the prediction of speciation for two synthetic experimental mixture spectra. The proposed model and the dataset generation strategies are generalized and can be implemented for other gases, different frequency ranges, and spectroscopy types. The multi-component speciation method developed herein is the first application of a convolutional neural network model, trained on HITRAN-based simulations, for spectral identification.

## 1. Introduction

As a powerful method for quantitative gas detection, infrared (IR) or vibrational absorption spectroscopy has been the subject of significant research, and an extensive database of spectral lines for compounds of interest has been documented [1,2,3]. Trace gas detection using IR absorption spectroscopy has been applied in industrial process control [4], pollution environmental monitoring [5], combustion characterization [6], remote sensing [7,8], and earth observation systems [9,10]. Many of the applications of IR absorption spectroscopy for gas sensing have focused on detecting single compounds within a gaseous environment, where the presence of that compound is anticipated in advance. However, many applications would benefit from optical sensors that could simultaneously characterize complex mixtures of unknown speciation. The availability of a large number of fingerprint spectral features in the mid- and far-IR regions allows for the simultaneous detection of many compounds [1]. When coupled with machine learning methods, IR fingerprint spectroscopy offers potential for automated sensors for speciation in complex gas mixtures.

The prior literature has established the role of IR absorption spectroscopy in industrial and environmental gas sensing [11,12]. In recent work, conventional machine learning and statistical learning models, such as fully connected neural networks, support vector machines, random forests, and adaptive boosted trees, have have been developed for automated gas speciation [13,14,15,16]. Deep learning has recently been applied to multi-component gas speciation and concentration predictions [17,18]. The emergence of powerful deep learning pattern recognition in gas speciation applications, as explored here, is expected to benefit IR absorption gas sensing by replacing manual feature selection and spectral interpretation with automated processes [19,20]. Using these learning methods, sensors can learn on large quantities of spectral information (features and their shapes) in frequency bands that are arbitrarily selected and/or imposed by hardware or other constraints and make useful predictions on constituent species and their concentrations [17,18,21,22,23,24,25]. Automated feature extraction, based on compound-distinct spectral fingerprints, followed by deep neural network classification provides a model architecture that can automate gas sensing and supplant the limitations of expert humans in spectral feature selection and spectral interpretation.

In prior work, we have shown that support vector machines and ensemble methods, such as random forests, can achieve high accuracy for the classification of pure compounds from spectral signatures [14] but that deep learning approaches have advantages in performance for speciation in complex mixtures [17], due to their capabilities in feature extraction. In this paper, we propose a one-dimensional deep convolutional neural network model for gas classification based on IR absorption spectra. The model is applied to the detection of ten IR-active compounds of interest in mixtures containing up to three components. The compounds selected for study are of interest in the atmosphere and in industrial processes and have well-documented IR transitions [1]. They include water vapor, carbon dioxide, ozone, nitrous oxide, carbon monoxide, methane, nitric oxide, sulfur dioxide, nitrogen dioxide, and ammonia, all of which have been the subject of prior IR gas sensor development (e.g., [26,27,28,29,30,31,32]).

A simulated dataset of IR absorption spectra (400–4000 cm^−1^) for mixtures of the molecules of interest was generated and used for model training. Simulated spectra containing added noise were used for model testing. To illustrate the usefulness of the model in the context of gas sensing applications, frequency subregions were also examined, and speciation for synthetic experimental spectra was demonstrated. The deep convolutional neural network architecture takes input mixture absorption spectra. Convolutional and pooling operations are used to extract features [33]. Following feature extraction, classification is performed in a fully connected deep neural network. The architecture allows user-defined frequency range and thermodynamic conditions. It is also scalable in terms of the number of compounds considered, as long as the spectroscopic data or reference spectra are available, and the complexity of mixtures. The performance of the model is evaluated based on classification accuracy, precision, recall, and F1 score metrics. Lastly, model predictions are examined using Grad-CAM [34] (gradient-weighted class activation mapping) offers activation maps to isolate relevant frequency regions which play a strong role in classification. Class activation maps thus assist in frequency selection for cross-sensitive gas sensor design.

## 2. Method

**Figure 1:**
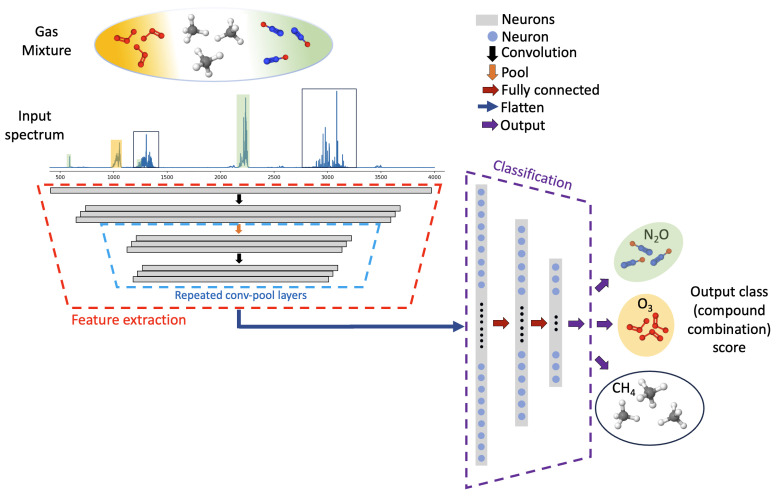
Schematic of the deep one-dimensional convolutional neural network infrared spectra classifier.

An overview of the deep learning classifier for infrared spectrum classification, developed herein, is shown in Figure 1. Absorption spectra, containing up to three absorbers diluted in air, from ten possible species, are simulated and used for training the deep learning model. Spectral absorption is defined by the Beer–Lambert law as shown below:

(1) $$\frac{I}{I_{0}}=exp\left(-A_{mixture}\right)=exp\left(-\sum \epsilon_{i}c_{i}l\right)$$

where *I* and $I_{0}$ are the transmitted and incident intensities, respectively, at the frequency of interest; $A_{mixture}$ is the absorbance for the mixture, which is the input to the deep neural network; $\epsilon_{i}$ and $c_{i}$ are the spectral absorption coefficient and concentration, respectively, of the *i*-th species in the gas mixtures; and *l* is the absorption pathlength.

During training, the model applies repeated convolution and pooling blocks to extract unique spectral information and downsample that information to reduce the dimensionality of the inputs to each layer of the network. The feature extraction and downsampling operations reduce the dimensionality of the problem from the original input absorbance values to a subset of extracted features. Following the feature extraction block, a flattening operation yields a single vector which enters a classification block containing three fully connected layers. The output layer of the classification block contains 175 neurons, each corresponding to a unique gas mixture or class. The model ultimately produces an integer output, providing prediction of the gas mixture, based on the input absorbance spectra.

### 2.1. Problem Formulation, Model Architecture, and Solution Approach

We consider the identification of components in a gas mixture by matching features in an unknown absorption spectrum to features in known reference spectra as a multi-label supervised classification problem. Hence, multiple labels are attributed to each spectra, one for each of the possible gaseous compounds present. We convert this multi-label classification problem to a multi-class classification problem using the label powerset method [35].

For the full wavenumber (frequency) range considered, 400–4000 cm^−1^ (wavelength of 2.5–25 μm) at 1 cm^−1^ resolution, the input absorbance is represented by a vector $x\in R^{n}$ (n=3601, *n* is vector length). Smaller ranges were also considered: 500–2000 cm^−1^ (5–20 μm, n=1501); 1000–1500 cm^−1^ (6.67–10 μm, n=501); 1250–1500 cm^−1^ (6.67–8 μm, n=251); 1000–2000 cm^−1^ (5–10 μm, n=1001); and 2000–4000 cm^−1^ (2.5–5 μm, n=2001). Spectra for the smaller wavenumber ranges were considered at a resolution of 1 cm^−1^ and, hence, have smaller input absorbance vectors (length *n*) than the larger range. The model is flexible and allows for user-defined wavenumber ranges.

Each input absorbance vector is associated with a class-indicator integer y∈R, where, *y* is an integer from 0 to 174 representing a unique mixture composition. Mixtures containing one (pure), two, and three components are considered in this study. A deep neural network classification model is developed to obtain an approximate learned hypothesis function, *h*, relating the spectrum to its class-indicator integer:

(2) h:x→y

In this multi-class classification problem, the last layer of the deep neural network is activated using the softmax function

(3) $$\mathrm{Softmax}\left(z_{i}\right)=\frac{e^{z_{i}}}{\sum_{j=1}^{k}e^{z_{j}}}$$

where $z_{i}$ represents the raw score or logit for a specific class (unique gas mixture), *i*. It is also the input to the softmax layer in the neural network (last layer). The numerator is the exponential of the raw score for class *i*. The denominator is the sum of the exponentials of raw scores for all *k* classes (unique combinations of mixture components). The softmax score is compatible with cross-entropy loss, offers stability in terms of model training, and highly penalizes the deep neural network for incorrect classifications.

The feature extraction block of the neural network (highlighted with dashed red lines in Figure 1) applies repeated convolution and pooling operations to reduce the high-dimensional input to the network by capturing the component-distinctive spectral features. The convolution operation involves traversing a sliding window, a randomly initialized filter kernel, over its inputs, which captures important spectral feature information while marginally reducing the dimensionality. Each pooling layer substantially downsamples its inputs. The weights of the convolutional and pooling layers are trained by passing the input spectrum through each of these layers during a forward training pass. Then, a loss function is evaluated, and weights are updated during the backward propagation of error [36]. The size of the input absorbance vector varies based on the frequency range; thus, the number of convolution and pooling blocks is varied to ensure that there are a sufficient number of neurons in the final layer to perform classification.

Each convolution converts the input vector to a new vector whose size is given by $\frac{1}{S}\left(W-K_{s}+2P\right)+1$, where *W*, $K_{s}$, *P*, and *S* are the size of the input, kernel, padding, and the stride, respectively. We found three filters provided good classification performance and did not substantially reduce the input dimensionality in each layer. Consequently, for the full wavenumber range (400–4000 cm^−1^), the output of the first convolutional layer with three filters is given by $\frac{1}{1}\left(3601-3+2\times 0\right)+1=3599\times 3$, where the sizes of the input vector, kernel, and stride are 3601, 3, and 1, respectively, and valid (zero) padding was used. Each convolutional layers is followed by a pooling layer, where the output size is $\frac{\left(W-K_{s}+1\right)}{S}$. For a stride size of two and a pooling kernel size of two, the total output shape is 1799×3 for the full range (400–4000 cm^−1^). For details of convolution and pooling operations, see [37].

The feature extraction block produces a matrix, due to the application of multiple filter kernels, containing the most relevant spectra-distinctive information from the input spectrum. This matrix is flattened to a vector and sent to a fully connected dense neural network (highlighted with dashed blue lines in Figure 1) for classification. Each dense layer applies learned weights and biases followed by nonlinear ReLU activation. Here, the Adam optimizer was implemented to update weights and biases using a sparse categorical cross-entropy loss function [38]. A batch size of 32 was used for training, and the network was trained for 40 epochs. With the application of a softmax layer, the final output from the neural network is a vector containing 175 softmax scores. The highest score indicates the class (mixture components) predicted by the neural network. The prediction is then processed to produce classification metrics for the assessment of model performance.

The final CNN architecture was selected through a grid-search hyperparameter tuning process, as outlined in our prior work [22]. The architecture is flexible, with the user-defined and variable input frequency bands (variable input vector lengths) requiring variable degrees of downsampling in the feature extraction block to extract a low-dimensional representation of the input spectrum. A pool size and kernel size of 2 were chosen to halve the input feature space, controlling the depth of the feature extraction block in the network. Through hyperparameter tuning, the optimal number of filters and kernel size for convolutional layers was found to be 3. The tuning process revealed that adding a hidden layer between the output of the last convolutional layer and the final classification output layer improves classification accuracy. To enable Grad-CAM, a convolutional layer with trainable weights is essential. Therefore, a convolutional layer is consistently added before the reduced low-dimensional feature space undergoes classification in the fully connected dense neural network.

The classification model is implemented in Python version 3.10.12 using the following library packages: TensorFlow version 2.15, NumPy version 1.25, Pandas version 1.5, and scikit-learn version 1.2. The model code is executed on an Intel Xeon CPU with a clock speed of 2 GHz and 12 GB RAM. The training time for 40 epochs are approximately 20 min if the model is trained across the entire frequency region. Inference on the entire test spectra takes approximately 14 s.

### 2.2. Spectra Simulation for Training, Validation, and Testing

**Figure 2:**
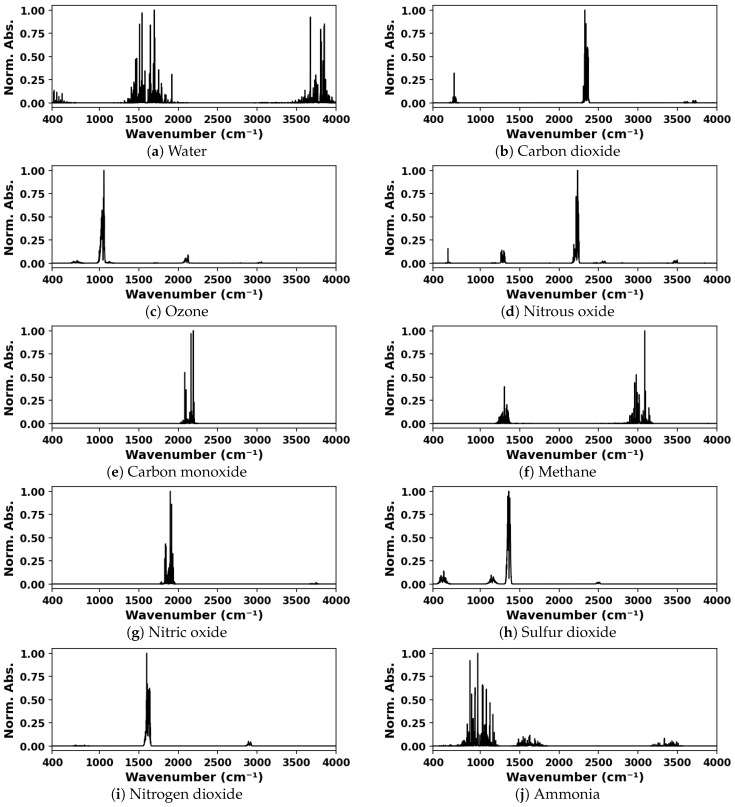
Representative simulated spectra (normalized absorbance vs. wavenumber); conditions: 1 atm, 297 K, absorber dilute in air.

Simulated IR absorption spectra were used for training and validation of the deep neural network. Spectra for mixtures containing one, two, or three components, from a possible ten species, were generated from spectral lines found in the HITRAN database [1] using the HAPI tool [39]. The ten species considered were water vapor, carbon dioxide, ozone, nitrous oxide, carbon monoxide, methane, nitric oxide, sulfur dioxide, nitrogen dioxide, and ammonia. The spectra were generated for a standard thermodynamic condition, 297 K and 1 atm total pressure, and for a 10 cm absorption pathlength. The model was trained on various ranges of spectral data including and within the 400–4000 cm^−1^ wavenumber range at a resolution of 1 cm^−1^. The representative simulated spectra for each species considered are shown in Figure 2.

**Figure 3:**
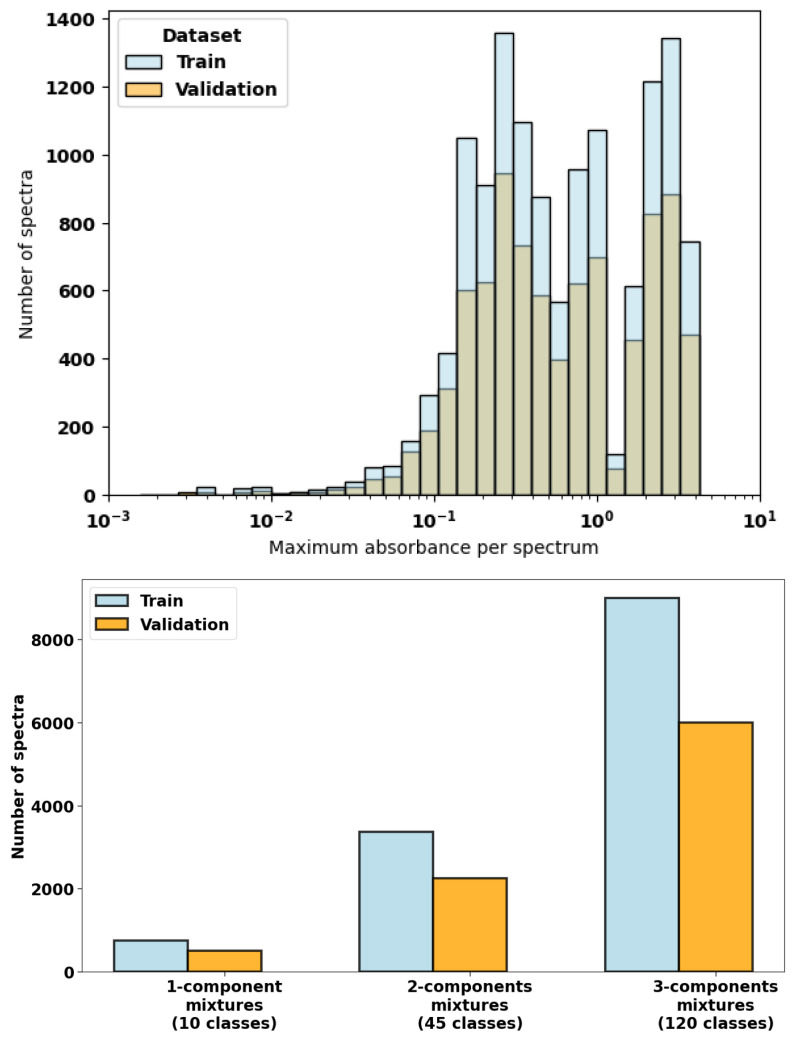
Distribution of spectra in training and validation datasets by maximum absorbance in each spectrum (400–4000 cm^−1^) (**top**) and number of components in the mixture (**bottom**).

For the ten species considered, there are 120 unique 3-component mixtures (^10^$C_{3}=120$), 45 2-component mixtures (^10^$C_{2}=45$), and 10 1-component (pure in air) mixtures. To produce training and validation datasets that are balanced, not favoring mixtures containing a larger number components, a larger number of discrete concentrations were considered for mixtures containing fewer components. Hence, for 3-component gas mixtures, we considered 5 discrete concentrations; for 2-component mixtures, we considered 12 discrete concentrations; and for 1-component mixtures, we considered 125 discrete concentrations (see Table 1). Mixture spectra were obtained by a linear combination of pure gas spectra in air at 1 atm, which assumes that all collisional broadening is air dominated. To obtain a balanced set of training spectra, spectra were randomly sampled from each of the unique combinations of species to generate 15,000 3-component spectra, 5625 2-component spectra, and 1250 1-component spectra (21,875 total spectra). This set of simulated spectra was divided into training (13,125 spectra) and validation (8750 spectra) datasets using a stratified 60–40% split. The distribution of spectra in each dataset is shown in Figure 3. The concentrations of absorbing gases considered in the dataset, given in Table 1, were specified at mole fractions that are relevant to industrial and atmospheric gas sensing conditions. The maximum absorbance for each spectrum ranges from $10^{-3}$ to 4, which was chosen as a reasonable range for practical gas sensing applications. See Figure 3 for a histogram of the maximum absorbance in the spectra contained in the training and validation datasets.

**Figure 4:**
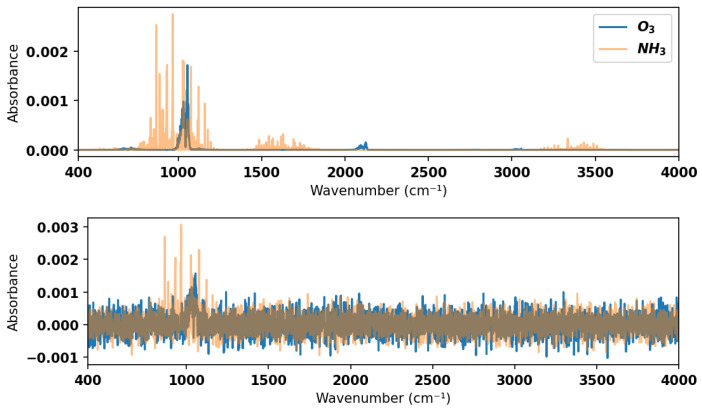
Example of noise-free (**top**) and noisy (**bottom**) spectra for 10 ppm ozone and 10 ppm ammonia dilute in air at 297 K and 1 atm; absorption pathlength of 10 cm. In practice, the model considers the composite spectrum which is the summation of the contributions from ozone and ammonia. Here, they are shown in different colors for ease of visual discrimination of the species contributions. The model reported here correctly identifies this noisy spectrum.

To ensure sufficient testing of the model, a test dataset comprising simulated spectra with added noise was generated. Noise in the experimental gas sensor signal is typically of the order of $10^{-3}$ or less in absorbance for direct absorption measurements without filtering or other efforts to improve the signal-to-noise ratio. Hence, we added random Gaussian noise with an amplitude of $10^{-3}$ in absorbance to the simulated mixture spectra contained in the validation dataset to form the test dataset. See Figure 4 for an example of a noisy spectrum for a 2-component mixture with comparison to the noise-free equivalent. The model was tested against this noisy test dataset in the large wavenumber range in which it was trained (400–4000 cm^−1^) as well the smaller ranges, which are more indicative of practical spectrometers. In the smaller wavenumber ranges, some species have a maximum absorbance below the $10^{-3}$ absorbance level and hence are always undetectable, irrespective of the classification method. Those species have not been considered in the performance evaluation for smaller wavenumber ranges.

## 3. Results and Discussion

### 3.1. Model Training and Validation

**Figure 5:**
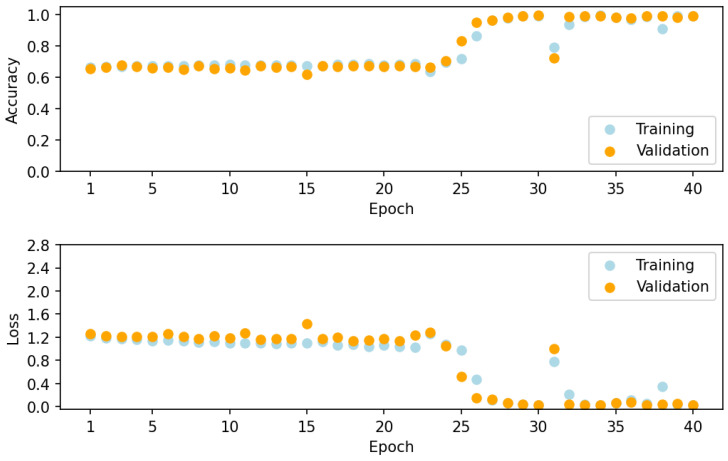
Model loss and accuracy on training and validation spectra in the 400–4000 cm^−1^ range.

Figure 5 illustrates the loss and overall accuracy during model training and validation. Due to the presence of a large number of relatively weak absorption spectra, through approximately 25 epochs, the accuracy is around 65%. After approximately 25 epochs, the loss is significantly reduced and the model approaches higher accuracy, ultimately achieving an overall accuracy of greater than 99% on the validation dataset after 40 epochs, at which point training is stopped.

### 3.2. Model Testing and Performance

**Figure 6:**
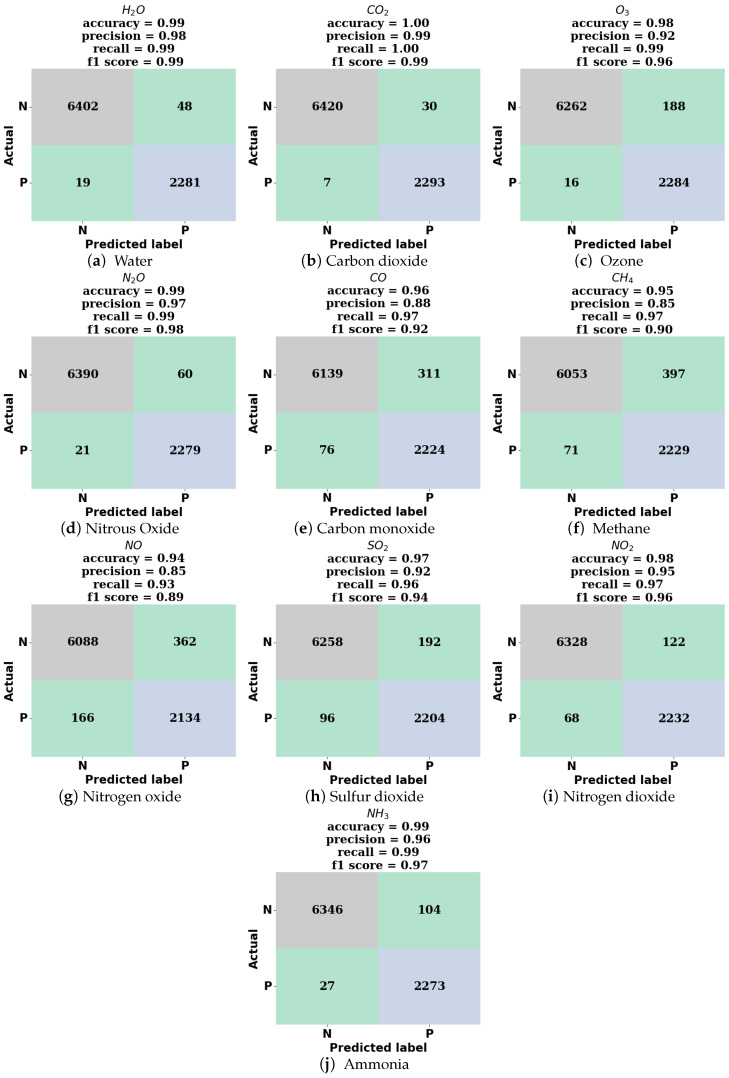
Confusion matrices for model predictions on noisy test spectra in the 400–4000 cm^−1^ range. N is negative and P is positive. Upper left box in matrices: true negative; upper right: false positive; lower left: false negative; lower right: true positive.

With the trained model achieving high accuracy on the validation dataset, the model was next interrogated for the prediction of the noisy test dataset in the IR range of 400–4000 cm^−1^ (2.5–25 μm). In this large wavenumber range, containing many fingerprinting features, the addition of noise only slightly reduces model performance with the model achieving an overall accuracy of 97%, precision of 93%, recall of 98%, and F1 score of 95%. See Figure 6 for confusion matrices which illustrate the model predictions on the noisy test dataset (8750 spectra) and Table 2 for tabulated performance metrics for all species in this wavenumber range.

Next, the model performance was investigated for mixture speciation, using the noisy test dataset, in several smaller wavenumber ranges: 500–2000 cm^−1^ (5–20 μm), 1000–1500 cm^−1^ (6.67–10 μm), 1250–1500 cm^−1^ (6.67–8 μm), 1000–2000 cm^−1^ (5–10 μm), and 2000–4000 cm^−1^ (2.5–5 μm). In these cases, only noisy test spectra in the smaller wavenumber ranges of interest were input to the model for evaluation. The results are shown in Table 3, Table 4, Table 5, Table 6 and Table 7, where the accuracy, precision, recall, and F1 scores for the classification of compounds present in each of the 8750 test spectra in each wavenumber range are tabulated. The model performs generally quite well against the noisy test dataset in the smaller ranges with overall accuracy in the range of 82% to 97%. It is important to note that if a species has no absorption lines in a particular wavenumber range or a maximum absorbance in the range less than $10^{-3}$, below the noise floor, then that species cannot be detected in that frequency band. Here, we do not consider cases where a species produces less than a maximum absorbance of $10^{-3}$ within a particular frequency band and label those cases as “N/A” in Table 2, Table 3, Table 4, Table 5, Table 6 and Table 7.

In the smaller frequency ranges, the model performance is reduced in instances where the strongest fingerprinting absorption features are not present in the considered frequency band. For example, nitrous oxide has a strong absorption feature around 2200 cm^−1^ and in the large band (400–4000 cm^−1^) is exceptionally well identified (99% accuracy and 99% recall). Nitrous oxide also has a a weaker feature around 1300 cm^−1^. When considering smaller frequency ranges where this weaker feature is used for nitrous oxide detection, the model performance is reduced; e.g., nitrous oxide classification has 76% accuracy and 52% recall for 1000–1500 cm^−1^ and 95% accuracy and 82% recall for 1250–1500 cm^−1^.

Other instances of misclassification occur in the smaller frequency ranges when there is not a sufficient spectral fingerprint for the model to separate two species with features at similar frequencies. For example, in the 1000–1500 cm^−1^ range, the model has slightly lower performance for the noisy test dataset due to there being spectral overlap between nitrous oxide and methane and ozone and ammonia (illustrated in Figure 4). However, the performance metrics illustrate that the model generally handles these cases well and only has difficulties separating overlapping spectral features when those features are of the same absorbance magnitude or are near the noise floor.

### 3.3. Class Activation Maps-Based Interpretability

Grad-CAM (gradient-weighted class activation mapping) offers a visual indication of how a convolutional neural network prioritizes different parts of the input by examining how gradients in the local input flow through the network itself, in particular to the last convolutional layer [34]. Grad-CAM produces heat maps which indicate the importance of different frequencies (spectral features) that are prioritized by the model for predicting speciation. Grad-CAM highlights features that the deep learning model prioritizes for classification, where bright regions in the grad-CAM heat map indicate a region or feature that positively contributes toward the classification decision.

**Figure 7:**
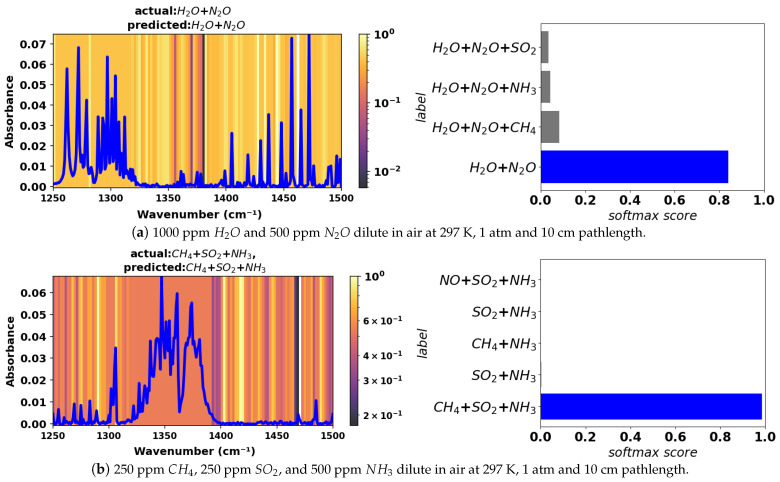
Grad-CAM heat maps for example noisy test spectra in the 1250–1500 cm^−1^ range. The bar plots next to each spectrum show softmax scores predicted by the model and the corresponding mixture constituents.

See Figure 7 for an example of grad-CAM heat maps for a two- and a three-component mixture. In the two-component example for H_2_O and N_2_O diluted in air (top graph), N_2_O features exist at wavenumbers less than 1320 cm^−1^, and H_2_O features exist at wavenumbers from 1350 to 1500 cm^−1^. The heat map shows the model prioritizing several spectral lines for both species within their respective absorption ranges for the positive identification of the two species that are present. For the three-component example containing CH_4_, SO_2_, and NH_3_ diluted in air (bottom graph), the spectrum contains a number of isolated CH_4_ lines from 1250 to 1310 cm^−1^, broad band SO_2_ absorption from 1320 to 1400 cm^−1^, and several weak but isolated NH_3_ lines from 1450 to 1500 cm^−1^. The heat map shows that the model prioritizes several specific locations where the isolated CH_4_ and NH_3_ lines exist, allowing a postive identification of these species. The broad SO_2_ absorption feature has a constant heat map color, indicating that the neural network is treating the signal from wavenumbers within this broad feature equally for the purpose of classification (i.e., the model has identified this as a broad feature from one species for the positive identification of SO_2_). Note that the softmax scores are relatively high for both spectra, indicating that the model assigns a high probability (confidence) to its predictions.

### 3.4. Experimental Demonstration

**Figure 8:**
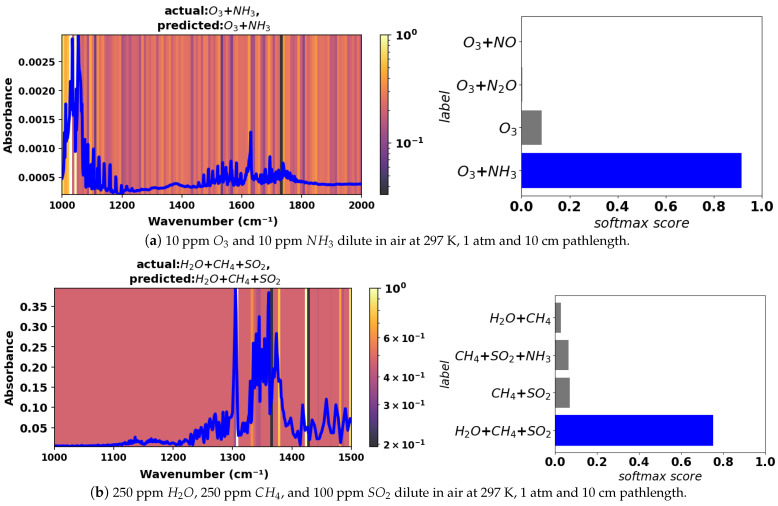
Grad-CAM heat maps for synthetic experimental mixture spectra in the 1000–2000 cm^−1^ and 1000–1500 cm^−1^ ranges. The bar plots next to each spectrum show softmax scores predicted by the model and the corresponding mixture constituents.

To demonstrate the model against experimental data, two synthetic experimental spectra were constructed from experiments for pure compounds reported in the National Institute of Standards and Technology (NIST) molecular spectroscopy database [40,41]. The experimental spectra were formed by concentration-weighted linear mixing of pure experimental spectra from that database. See Figure 8 for these two spectra, Grad-CAM heat maps, and the corresponding model mixture prediction softmax scores. One mixture of spectra comprises ozone and ammonia in the 1000–2000 cm^−1^ range. The other mixture of spectra is comprises water vapor, methane, and sulfur dioxide in the 1000–1500 cm^−1^ range. The model predicts the correct mixtures based on these spectra with a high degree of confidence, as shown by the softmax scores.

For the ozone–ammonia mixture, the model prioritizes absorption lines near 1070 cm^−1^ for classification of ozone. Lines from 1450 to 1750 cm^−1^, as well as some lines from 1150 to 1200 cm^−1^, allow the model to correctly classify ammonia. For the water vapor–methane–sulfur dioxide mixture in the 1000–1500 cm^−1^ range, the model prioritizes a strong feature around 1300 cm^−1^ for the classification of methane. Sulfur dioxide is classified by the model using features around 1330 and 1380 cm^−1^. And the model prioritizes features from 1400 to 1500 cm^−1^ for the classification of water vapor.

## 4. Conclusions

A one-dimensional convolutional deep learning neural network classification model has been developed and demonstrated for the automated speciation of gaseous mixtures based on their infrared (IR) absorption spectra. The model has been demonstrated for the speciation of mixtures containing up to three components and for the detection of ten gaseous compounds relevant to industrial and environmental processes and sensing applications: water vapor, carbon dioxide, ozone, nitrous oxide, carbon monoxide, methane, nitric oxide, sulfur dioxide, nitrogen dioxide, and ammonia. The model achieved high accuracy of greater than 99% on a noise-free validation dataset and an accuracy of 97% on a noisy test dataset (8750 spectra) in a large region of 400–4000 cm^−1^. The model was examined in several smaller IR bands of interest and shown to perform well with overall accuracy in the range of 82% to 97%. Additionally, the model was demonstrated in smaller bands for the speciation of mixtures from synthetic experimental spectra. Class activation maps illustrate how the model prioritizes spectral features for classification.

The model can be applied in gas sensing scenarios where the gas mixtures are complex and/or of unknown speciation. The model is highly scalable, allowing for the consideration of any gas with known spectroscopic parameters or a known reference spectrum and for gas mixtures containing any number of components. The model can also be implemented in different frequency regions, for different thermodynamic conditions, and for classification using different types of spectroscopy, so long as suitable training datasets exist or can be generated. Future work will focus on extending the methods developed here for determination of quantitative concentrations, improved sensitivity, and speciation in more complex systems (increased number and mixtures components and species that are detectable).

## Figures and Tables

**Table 1:** Component concentrations for simulated mixture spectra, in parts per million (ppm) diluted in air. For 2-component mixtures, 12 concentrations are evenly spaced within the given ranges. For 1-component mixtures, 125 concentrations are evenly spaced within the given ranges.

Compound	3-Component Mixtures (ppm)	2-Component Mixtures (ppm)	1-Component Mixtures (ppm)
H_2_O, water vapor	1000, 5000, 10,000, 15,000, 20,000	1000–20,000	1000–20,000
CO_2_, carbon dioxide	100, 500, 1000, 1500, 2000	100–2000	100–2000
O_3_, ozone	10, 250, 500, 750, 1000	10–1000	10–1000
N_2_O, nitrous oxide	10, 250, 500, 750, 1000	10–1000	10–1000
CO, carbon monoxide	10, 250, 500, 750, 1000	10–1000	10–1000
CH_4_, methane	10, 250, 500, 750, 1000	10–1000	10–1000
NO, nitric oxide	10, 250, 500, 750, 1000	10–1000	10–1000
SO_2_, sulfur dioxide	10, 250, 500, 750, 1000	10–1000	10–1000
NO_2_, nitrogen dioxide	10, 250, 500, 750, 1000	10–1000	10–1000
NH_3_, ammonia	10, 250, 500, 750, 1000	10–1000	10–1000

**Table 2:** Accuracy, precision, recall, and F1 scores for classification of noisy test spectra in the 400–4000 cm^−1^ range.

Metric	H_2_O	CO_2_	O_3_	N_2_O	CO	CH_4_	NO	SO_2_	NO_2_	NH_3_	Overall
Accuracy	0.99	1.00	0.98	0.99	0.96	0.95	0.94	0.97	0.98	0.99	0.97
Precision	0.98	0.99	0.92	0.97	0.88	0.85	0.85	0.92	0.95	0.96	0.93
Recall	0.99	1.00	0.99	0.99	0.97	0.97	0.93	0.96	0.97	0.99	0.98
F1 score	0.99	0.99	0.96	0.98	0.92	0.90	0.89	0.94	0.96	0.97	0.95

**Table 3:** Accuracy, precision, recall, and F1 scores for classification of noisy test spectra in the 500–2000 cm^−1^ range.

Metric	H_2_O	CO_2_	O_3_	N_2_O	CO	CH_4_	NO	SO_2_	NO_2_	NH_3_	Overall
Accuracy	0.97	0.95	0.94	0.94	N/A	0.91	0.89	0.95	0.95	0.97	0.94
Precision	0.90	0.88	0.99	0.86	N/A	0.77	0.74	0.89	0.94	0.92	0.87
Recall	0.99	0.91	0.79	0.94	N/A	0.92	0.92	0.93	0.88	0.97	0.92
F1 score	0.94	0.90	0.88	0.90	N/A	0.84	0.82	0.91	0.91	0.95	0.89

**Table 4:** Accuracy, precision, recall, and F1 scores for classification of noisy test spectra in the 1000–1500 cm^−1^ range.

Metric	H_2_O	CO_2_	O_3_	N_2_O	CO	CH_4_	NO	SO_2_	NO_2_	NH_3_	Overall
Accuracy	0.88	N/A	0.84	0.76	N/A	0.74	N/A	0.84	N/A	0.86	0.82
Precision	0.74	N/A	0.76	0.57	N/A	0.52	N/A	0.73	N/A	0.75	0.67
Recall	0.89	N/A	0.61	0.52	N/A	0.69	N/A	0.66	N/A	0.70	0.68
F1 score	0.80	N/A	0.67	0.54	N/A	0.59	N/A	0.69	N/A	0.73	0.67

**Table 5:** Accuracy, precision, recall, and F1 scores for classification of noisy test spectra in the 1250–1500 cm^−1^ range.

Metric	H_2_O	CO_2_	O_3_	N_2_O	CO	CH_4_	NO	SO_2_	NO_2_	NH_3_	Overall
Accuracy	1.00	N/A	N/A	0.95	N/A	0.94	N/A	0.96	N/A	0.90	0.95
Precision	1.00	N/A	N/A	1.00	N/A	0.96	N/A	1.00	N/A	0.98	0.99
Recall	1.00	N/A	N/A	0.82	N/A	0.83	N/A	0.84	N/A	0.65	0.83
F1 score	1.00	N/A	N/A	0.90	N/A	0.89	N/A	0.91	N/A	0.78	0.90

**Table 6:** Accuracy, precision, recall, and F1 scores for classification of noisy test spectra in the 1000–2000 cm^−1^ range.

Metric	H_2_O	CO_2_	O_3_	N_2_O	CO	CH_4_	NO	SO_2_	NO_2_	NH_3_	Overall
Accuracy	1.00	N/A	0.98	0.97	N/A	0.94	0.96	0.99	0.99	0.97	0.97
Precision	0.99	N/A	0.95	0.91	N/A	0.84	0.90	0.98	0.96	0.93	0.93
Recall	1.00	N/A	0.99	0.97	N/A	0.96	0.94	0.98	1.00	0.97	0.98
F1 score	0.99	N/A	0.97	0.94	N/A	0.89	0.92	0.98	0.98	0.95	0.95

**Table 7:** Accuracy, precision, recall, and F1 scores for classification of noisy test spectra in the 2000–4000 cm^−1^ range.

Metric	H_2_O	CO_2_	O_3_	N_2_O	CO	CH_4_	NO	SO_2_	NO_2_	NH_3_	Overall
Accuracy	0.97	0.98	0.85	0.97	0.95	0.94	N/A	0.87	0.89	0.82	0.92
Precision	0.93	0.94	0.68	0.93	0.91	0.90	N/A	0.72	0.75	0.60	0.80
Recall	0.98	0.98	0.80	0.97	0.89	0.88	N/A	0.86	0.85	0.88	0.90
F1 score	0.95	0.95	0.74	0.95	0.90	0.89	N/A	0.78	0.80	0.72	0.85

## Data Availability

Code and data will be made available upon reasonable request.

## Abbreviations

The following abbreviations are used in this manuscript:

IR	infrared
PPM	parts per million
